# Magnesium supplementation and iron status among female students: The intervention study

**DOI:** 10.5937/jomb0-33898

**Published:** 2022-07-29

**Authors:** Neda Milinković, Milica Zeković, Margarita Dodevska, Brižita Đorđević, Branimir Radosavljević, Svetlana Ignjatović, Nevena Ivanović

**Affiliations:** 1 University of Belgrade, Faculty of Pharmacy, Department of Medical Biochemistry; 2 University of Belgrade, Institute for Medical Research, National Institute of the Republic of Serbia, Center of Excellence in Research, Nutrition and Metabolism; 3 Institute of Public Health of Serbia "Dr Milan Jovanović Batut", Center for Hygiene and Human Ecology; 4 University of Belgrade, Faculty of Pharmacy, Department of Bromatology; 5 University of Belgrade, Faculty of Medicine, Institute of Chemistry in Medicine; 6 University Clinical Center of Serbia, Center for Medical Biochemistry

**Keywords:** magnesium, supplementation, iron status, female, students, magnezijum, suplementacija, status gvožđa, žene, studenti

## Abstract

**Background:**

Literature data indicate the benefit of magnesium (Mg) supplementation. The aim of this study was to examine the effect of short-term Mg supplementation on iron status in healthy female participants.

**Methods:**

One hundred healthy female students of the University of Belgrade - Faculty of Pharmacy participated the study during eleven intervention days. Students ingested Mg preparations with the same dose of the active substance. The analysis included the measurement of serum iron, unsaturated iron binding capacity (UIBC), total iron binding capacity (TIBC), total Mg (tMg), ionized Mg (iMg), complete blood count, met-, carboxyand oxy-haemoglobin (metHgb, COHgb, O2Hgb). Transferrin concentrations and percentage of transferrin saturation (SAT) were calculated manually. The association among the analyzed biochemical parameters was examined using polynomial regression. A principal component analysis (PCA) was used for the evaluation of interdependence between the analyzed parameters.

**Results:**

A statistically significant trend for change in O2Hgb (%) by tertiles of iMg concentrations was found (P = 0.029). Serum tMg reached significant positive correlation with the SAT at concentration levels greater than 0.9 mmol/L, after 11 days of intervention (R2=0.116). Ionized Mg in a concentration higher than 0.6 mmol/L is positively correlated with SAT and serum Fe (R2=0.214; 0.199, respectively). PCA revealed variability of 64.7% for two axes after 11 days.

**Conclusions:**

Mg supplementation leads to an improvement in the certain iron status parameters even in individuals with optimal levels of these indices. However, caution should be exercised when supplementing Mg, and laboratory monitoring of the interaction is required.

## Abstract (sr)

**Uvod:**

Literaturni podaci ukazuju na benefit suplementacije magnezijumom (Mg). Cilj ove studije bio je da se ispita uticaj kratkotrajne suplementacije Mg na status gvožđa kod zdravih žena.

**Metode:**

Sto zdravih studentkinja Univerzitet u Beogradu - Farmaceutskog fakulteta je učestvovalo u istraživanju tokom jedanaest dana intervencije. Studenti su uzimali preparate Mg sa istom dozom aktivne supstance. U serumu je određivano gvožđe, nezasićen kapacitet vezivanja gvožđa (UIBC), ukupan kapacitet vezivanja gvožđa (TIBC), ukupan Mg (tMg), jonizovni Mg (iMg), kompletna krvna slika, met-, karboksiioksihemoglobin (metHgb, COHgb, O2Hgb). Transferin i saturacija transferina (SAT) su izračunati ručno. Povezanost analiziranih biohemijskih parametara je ispitana pomoću polinomalne regresije. Za procenu međuzavisnosti između analiziranih parametara korišćena je analiza glavnih komponenti (PCA).

**Rezultati:**

Utvrđen je statistički značajan trend promene O2Hgb (%) po tertilima koncentracija iMg (P = 0,029). Ukupan Mg je dostigao značajnu pozitivnu korelaciju sa SAT pri koncentracijama većim od 0,9 mmol/L, nakon 11 dana intervencije (R2 = 0,116). Jonizovani Mg u koncentraciji većoj od 0,6 mmol/L pozitivno korelira sa SAT i gvožđem (R2 = 0,214; 0,199, redom). PCA analizom je pokazana varijabilnost od 64,7% za dve ose nakon 11 dana.

**Zaključak:**

Suplementacija Mg dovodi do poboljšanja određenih parametara statusa gvožđa čak i kod pojedinaca sa optimalnim nivoima ovih parametara. Međutim treba biti oprezan pri suplementaciji Mg, a dodatno je neophodno i laboratorijsko praćenje ovih interakcija.

## Introduction

Magnesium (Mg) plays an important role in many physiological functions. As a cofactor for over 600 enzymes and an activator of an additional 200 enzymes, Mg is involved in almost all major biochemical and metabolic processes in the body. Magnesium has an important role in macronutrient and energy metabolism, neuromuscular function, bone development, cell proliferation and signalling pathways [1] [2]. Magnesium is an essential micronutrient, and therefore it must be supplied regularly via food sources to reach the recommended intake and prevent deficiency. Insufficient dietary intake of Mg is one of the most important causes of hypomagnesaemia [3]. Re commendations for Mg intake for adults in the United States (Recommended Daily Allowances, RDA) are 320 mg/day for women and 420 mg/day for men and in Europe 300 mg/day for women and 350 mg/day for men (Dietary Reference Values, DRVs) [4] [5]. Although Mg is widely distributed in plant and animal foods as well as in beverages, literature data indicate that the dietary intake of Mg is below the recommended levels in a large percentage of European and US populations where there is a higher prevalence of the Western dietary pattern [6] [7] [8] [9]. There is also evidence that many young women in various European countries and in the US fail to achieve these recommended intakes [2] [10]. Furthermore, epidemiological studies have shown that people who follow a Western-style diet, characterized by a high intake of processed foods, have an inadequate intake of several micronutrients, with dietary Mg intake of less than 30-50% of RDA [11] [12].

In recent decades, insufficient Mg intake and consequent hypomagnesaemia have been associated with several cardiovascular conditions, including hypertension, an increased risk of glucose intolerance, metabolic syndrome, and type 2 diabetes [1] [2] [3] [13]. Moreover, a meta-analysis of eight prospective cohort studies has reported a significant inverse association between Mg intake and risk of type 2 diabetes in a dose-response manner [14]. In addition, Mg deficiency is closely related to the development of anaemia [15] [16].

There is accumulating research regarding the importance and modalities for achieving adequate Mg intake. Magnesium supplementation may be a good strategy for preventing deficiency and prevent-ing associated diseases, when the recommended daily intake cannot be provided solely by the diet [1] [2] [3] [13]. According to the recently conducted survey, Mg supplements were among ten most popular dietary supplements used in adult population [17]. Magne sium supplementation led to improved anemia in thalassemic mice and improved erythrocyte membrane transport abnormalities in patients with sickle cell disease [18] [19], while in athletes, Mg supplementation increased erythrocyte count and haemoglobin levels [20]. Moreover, according to the Shi et al. [16] Mg supplementation may be a safer alternative than iron (Fe) supplementation in the prevention of anemia.

Although previously published data suggest the beneficial effects of increased Mg intake on Fe status among anaemic individuals, research on this issue is lacking for the non-anaemic population [15] [16]. It is generally accepted that the female population is more prone to Fe deficiency anemia and more vulnerable on Fe status than men [21] [22]. Therefore, it is important to ensure stable Fe status, particularly in the reproductive period of life. The aim of this study was to examine the effect of short-term Mg supplementation in doses of 375 mg corresponding to the 100% of Mg Nutritive Referent Value NRV on Fe status in healthy female participants.

## Materials and methods

### Ethics statement

This study was conducted following the guidelines laid down in the Declaration of Helsinki and the study protocols were approved by the Ethics Commission of the University of Belgrade - Faculty of Pharmacy, Belgrade, Serbia (approval number: 188/2, 2020). All subjects went through verbal and written consent processes.

### Study design and subjects

One hundred healthy female students of the University of Belgrade - Faculty of Pharmacy agreed to participate in this study. Eligible students were approached in the Faculty setting. Recruitment brochure contained detailed information regarding the purpose of the study, procedures involved as well as rights and expectations of the potential participants. The main inclusion criteria were: age between 18 and 30 years, body mass index (BMI) between 18.5 and 29.9 kg/m^2^ and willingness to maintain regular dietary habits throughout the study. The study did not include students who had altered Fe status (primarily based on complete blood count analysis), who had taken Mg supplements over the previous three months, or students who had confirmed chronic kidney and/or gastrointestinal tract disease. Finally, after applying the exclusion criteria, 46 respondents remained in the analytical sample.

Anthropometric parameters were measured at the beginning of the study. Height was measured to the nearest 0.1 cm (Perspective Enterprises, Kalamazoo, MI, USA). Body weight and body fat percentage were determined using the bioimpedance method (BC-418MA, Tanita, USA). Body Mass Index (BMI) was calculated as weight (kg) / height^2^ (m^2^).

During eleven intervention days, students ingested Mg preparations (citrate, oxide and carbonate) with the same dose of the active substance (375 mg/day). The subjects were randomly assigned to three groups according to the form of the Mg preparation.

### Dietary intake assessment

Dietary intake data was collected via participants' subjective retrospective reports in two study points. Participants were administered 24h dietary recalls on two consecutive days before the initiation of the supplementation (t0) and after 11 days of using the provided Mg supplements, within the follow-up assessment (t2). Dietary information and relevant contextual data were obtained over the course of face-to-face structured interviews led by a trained researcher. In order to improve the accuracy of the report, enhance participants' memory, and assure the provision of detailed descriptions of consumed items multiple-pass methodological approach was applied. To assist respondents in portion size quantification interviewers used validated, 135-item Food Atlas featuring coloured photographs of increasing portion sizes for a selection of Balkan region-specific simple foods and composite dishes [23]. Additionally, subjects reported quantities of foods consumed based on standardized household measures, natural units and labelling information for packaged products. Diet Assess & Plan - original software-based platform for comprehensive nutritional assessment was applied in questionnaire processing [24]. Subsequent conversion of food consumption information into energy and nutrient intake estimates was performed according to data compiled in National Serbian Food Composition Database [25]. Age and gender-adjusted nutritional recommendations proposed by EFSA i.e., Dietary Reference Values (DRVs) were applied for micronutrient adequacy evaluation [26].

### Biochemical assessment

Serum biochemical parameters were analyzed before the initiation of the intervention, at baseline (t0), on the fifth day (t1) and the eleventh day (t2) of the intervention period. Blood samples were collected by professional phlebotomists via venipuncture between 7:00 am and 9:00 am. All studied participants were donated blood samples after the 12h of overnight fast. Standard operating procedures for blood collection and sample preparation were followed [27]. A closed venipuncture system, Beckton Dickinson (BD) 22 Standard Wire Gauge (SWG), a reusable adapter and vacutainers were used. Vacutainers with clot activator (BD Vacutainer® SST™ Tubes) were used to obtain serum samples. Serum samples were used for measurement of serum Fe, unsaturated iron binding capacity (UIBC), total iron binding capacity (TIBC) and total Mg (tMg). Transferrin concentrations and percentage of transferrin saturation (SAT) were calculated according to the formula proposed by Dacie et al. [28]. Complete blood count was determined using the whole blood samples collected in a tube with liquid anticoagulant (ethylenediaminetetraacetic acid, EDTA) (BD Vacutainer® EDTA Tubes). For the analyses of met-, carboxy- and oxy-haemoglobin (metHgb, COHgb, O_2_Hgb) and ionized Mg (iMg), lithium-heparin spray-coated tube (BD Vacutainer® Heparin Tubes) were used. Serum Fe, UIBC, TIBC and tMg concentrations were measured using the spectrophotometric method with commercial reagents, on Olympus AU400 biochemical analyzer (Beckman Coulter, Inc., California, USA). Complete blood count was measured in whole blood samples using electrical impedance on Coulter Ac•T diff Hematology Analyzer (Beckman Coulter, Inc., California, USA). Total haemoglobin (tHgb) concentrations were measured by spectrophotometry on Coulter Ac•T diff Hematology Analyzer. MetHgb, COHgb, O_2_Hgb and iMg were determined approximately up to 60 min after collection using Stat Profile Prime Plus Critical Care blood gas analyzer (Nova Biomedical, USA). The precision and accuracy of the methods were verified using commercial control samples for all the listed parameters.

### Statistical analysis

The normality of the data distribution was analysed using the One-sample Kolmogorov-Smirnov test. Normally distributed data were presented as mean and standard deviation (SD). Non-normally distributed data were presented as a median and interquartile range (IQR), by subtracting the first from the third quartile of the distribution. For data that were not normally distributed, values were log-transformed before analysis. Linear regression analysis was performed to evaluate the relationship between investigated parameters. The association among serum Fe and Mg (as tMg, iMg and iMg/tMg) with certain (some) biochemical parameters was presented as polynomial regression. A principal component analysis (PCA) was used for the evaluation of interdependence between serum Fe and Mg (as tMg, iMg and iMg/tMg) and biochemical parameters (UIBC, TIBC, Transferrin, tHgb, hematocrit, SAT and Fe). We have considered p-value < 0.05 as statistically significant. All statistical analyses were performed using IBM SPSS version 24 (SPSS Inc., USA).

## Results

Baseline participants' characteristics are presented in Table 1. Participants were on average 23 years old, with a BMI of 21.8 kg/m^2^. Erythrocyte indices and biochemical parameters indicated a favourable Fe status. All presented parameters were within the reference values routinely used in the laboratory, and recommended by the test manufacturer. Given that the aim of this study was to examine the effect of the applied dose of magnesium of 375 mg, in which magnesium is most often found in dietary supplements on the Serbian market (dose that meets 100% nutritional reference value for Mg), data was further analysed without partition of subjects into sub cohorts in relation to different types of supplemented magnesium (citrate, oxide and carbonate).

**Table 1:** Baseline participant characteristics (N=46). ^a^Mean±SD, the standard deviation for normal distribution<br>^b^Median (IQR); IQR, interquartile range (quartile3-quartile1) for not normally distribution WBC, white blood cells (Leucocytes); RBC, red blood cells; tHgb, haemoglobin; Hct, hematocrit; MCV, mean cell volume; MCH, mean haemoglobin concentration; MCHC, amount of haemoglobin per unit volume in a single red blood cell; Fe, iron; UIBC, unsaturated iron-binding capacity; TIBC, total iron-binding capacity; SAT, total transferrin saturation

Parameters	Mean±SD^a^ Median (IQR)^b^
Age, years	23 (2)
BMI, kg/m^2^	21.8 (2.8)
Total body fat, %	25.49±4.85
Systolic pressure (mmHg)	113.8±10.6
Diastolic pressure (mmHg)	80.3±8.6
WBC, 10^9^/L	7.29±1.13
Lymphocytes, %	33.5±5.7
Monocytes, %	6.0±1.6
Granulocytes, %	60.4±5.9
Lymphocytes, #	2.4±0.5
Monocytes, #	0.4 (0.1)
Granulocytes, #	4.4±0.9
RBC, 10^12^/L	4.51±0.28
tHgb, g/L	140 (4)
Hct, L/L	0.439±0.029
MCV, fL	89.6±4.2
MCH, pg	29.9±1.7
MCHC, g/L	334±7
RDW, %	14.5±1.7
iMg, mmol/L	0.59±0.032
tMg, mmol/L	0.89±0.054
Fe, μmol/L	14.89±5.65
UIBC, μmol/L	51.8±13.8
TIBC, μmol/L	66.7±12.5
SAT, %	23.9±9.8
Transferrin, g/L	2.6 (0.7)
MetHgb, %	0.4 (0.15)
COHgb, %	3.66±2.02
O_2_Hgb, %	40.6±19.7

Estimated daily energy and nutrient intakes are presented in Table 2. Based on the average of dietary recalls for two consecutive days at baseline evaluation (t0) and after 11 days (t2) of using provided dietary supplements, the results obtained indicate that food intake did not change over time.

**Table 2:** Daily energy and nutrient intakes among study participants assessed by the average of dietary recalls for two consecutive days at baseline evaluation (t0) and after 11 days (t2) of using provided dietary supplements. %TEI, percentage of total energy intake; p < 0.05 – statistically significant difference between t0 and t2 within the same intervention group.<br>^a^Mean±SD, the standard deviation for normal distribution

Intervention Group (N=46)			
Energy/Nutrients	t0	t2	P
Energy (kcal)	1733.6±550.6	1804.9±485.6	0.501
Carbohydrates (TEI%)	29.9±9.8	28.6±7.8	0.516
Proteins (%TEI)	10.5±3.9	11.7±3.3	0.066
Fats (%TEI)	29.0±13.1	31.9±13.1	0.283
Fe (mg)	7.8±3.3	8.6±3.0	0.177
Mg (mg)	236.2±85.1	230.8±74.1	0.741
Zn (mg)	7.3±3.3	8.4±3.2	0.052
Folic acid (μg)	189.2±96.6	210.9±78.2	0.269
Vitamin B12 (μg)	2.5±1.3	2.6±1.2	0.053
Vitamin C (mg)	61.5±7.3	68.9±6.0	0.631

Estimated intake levels of eight food groups and their corresponding contributions to daily iron intake based on repeated 24 h dietary recalls are presented in Table 3. Dominant Fe dietary sources were grains and cereal products (28.6%), meat and meat products (22.3%) and vegetables and vegetable products (15.7%).

**Table 3:** Daily intake levels presented as the median levels and 5th and 95th percentiles of eight food groups and their corresponding contributions to daily iron intake based on repeated 24 h dietary recalls among study participants.

Food groups	Intake of the food group<br>(mg/day)			The average contribution<br>to the total iron intake<br>(10.52 mg/day)	
	Median	5th<br>percentile	95th<br>percentile	%	Iron intake<br>(mg/day)
Milk and dairy products	0.18	0.01	0.44	2.25	0.24
Eggs and egg products	0.63	0.15	3.34	8.01	0.84
Meat and meat products	1.64	0.38	5.92	22.28	2.34
Grains and cereal products	2.28	0.71	5.60	28.57	3.00
Nuts and seeds	0.42	0.03	2.24	6.94	0.73
Vegetables and vegetable products	1.29	0.14	4.03	15.67	1.65
Fruit and fruit products	0.51	0.04	1.54	4.84	0.51
Sugar and confectionary products	0.21	0.01	1.95	4.37	0.46

A statistically significant trend for change in O_2_Hgb (%) by tertiles of whole blood iMg concentrations was found. With the increase of iMg, the percentage of O_2_Hgb decreases. Additionally, we found a significant increase in changes of SAT (%) by quartile until the third quartile of tMg values. Interestingly, in the fourth quartile of serum tMg values, a significant decrease in SAT (%) was observed.

**Figure 1:**
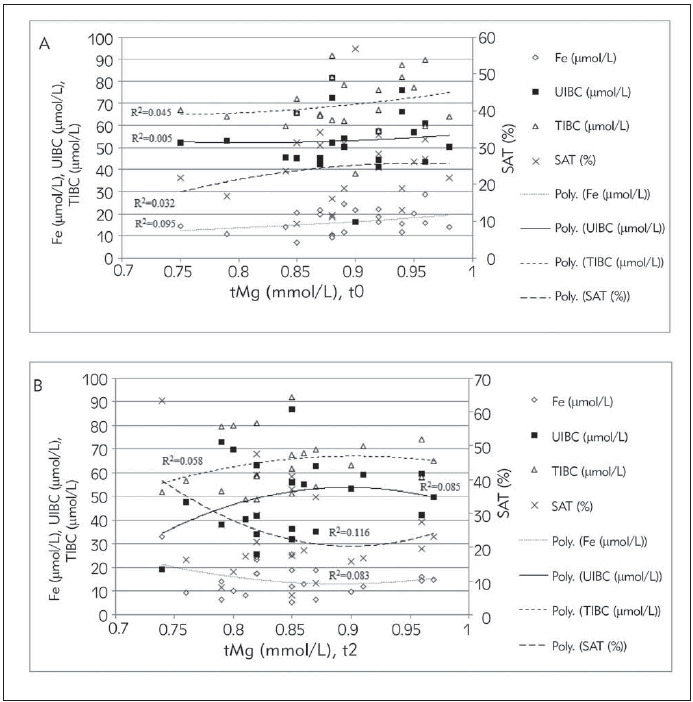
Interdependence between serum total magnesium (tMg) with serum iron (Fe),unsaturated iron binding capacity (UIBC), total iron binding capacity (TIBC) and transferrin saturation (SAT) at the beginning (A) and after 11 days (B) of supplementary intervention.

**Figure 2:**
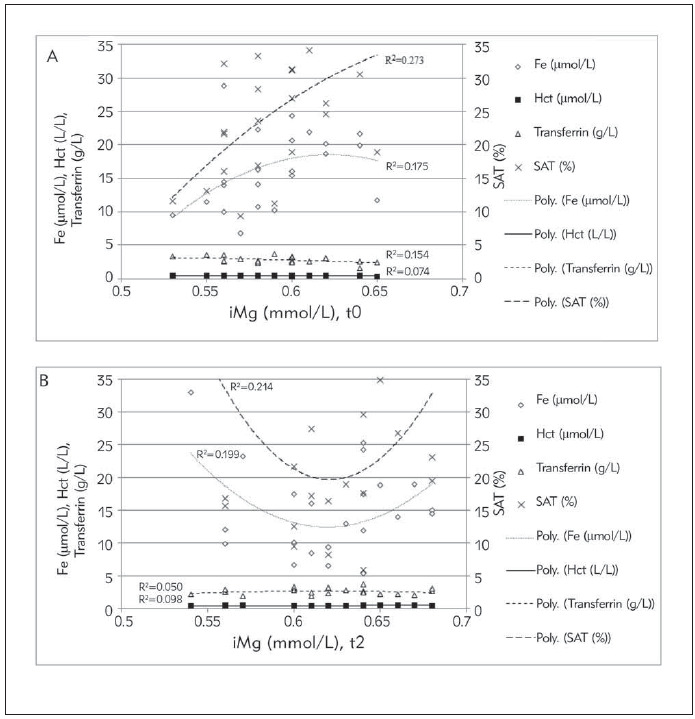
Interdependence between serum ionized magnesium (iMg) with serum iron (Fe), hematocrit (Hct), transferrin and transferrin saturation (SAT) at the beginning (A) and after 11 days (B) of supplementary intervention.

Based on polynomial regression analyses, serum tMg reached significant positive correlation with the SAT at concentration levels greater than 0.9 mmol/L, after 11 days of supplementary intervention (R^2^=0.116; Figure 1B). Before the intervention, there was no statistically significant correlation between serum tMg and SAT (Figure 1A). Ionized Mg in a concentration higher than 0.6 mmol/L is positively correlated with SAT and serum Fe (R^2^=0.214; 0.199, respectively; Figure 2B) after supplementation, which was not the case before the supplementation, because at the mentioned concentration the serum Fe decreased slightly (Figure 2A).

**Figure 3:**
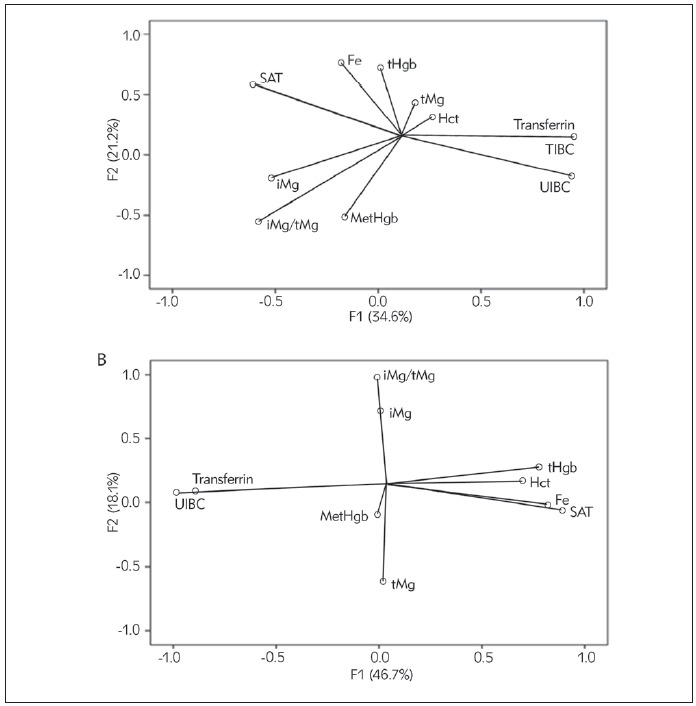
Principal Component Analysis for serum iron (Fe) and serum magnesum (Mg) (as total (tMg), ionized (iMg) and iMg/Mg) and biochemical parameters (unsaturated iron binding capacity (UIBC), total iron binding capacity (TIBC), transferrin, transferrin saturation (SAT), total haemoglobin (tHgb), MetHgb and hematocrit (Hct)) content at the beginning (A, t0) and after 11 days (B, t2) of supplementary intervention.

PCA was applied to integrate results of biochemical parameters, to discover the possible correlations among measured parameters, and to classify the parameters in a factor plane. PCA is a factor model in which the factors are based on summarizing the total variance. The first two factors should correspond to a high% of the variance to ensure that the maps based on the first two factors are a good quality projection of the initial multi-dimensional table. At the beginning of the experiment, PCA revealed that two axes participated in total variability with 55.8% (F1: 34.6% and F2: 21.2%), and after 11 days, the axes participated in total variability with 64.7% (F1: 46.7% and F2: 18.0%). According to the PCA, Transferrin, TIBC, UIBC, SAT, iMg/tMg and iMg correlated mainly with the first axis - factor (0.952; 0.952; 0.940; -0.607; -0.579 and -0.520, respectively), while serum Fe, tHgb, SAT, Mg; hematocrit; iMg/tMg, and MetHgb were mainly connected to the second axis - factor (0.758; 0.721; 0.588; 0.442; 0.317; -0.554; -0.510; respectively) Figure 3A. In the first factor (F1) there is a strong positive correlation between TIBC, UIBC and Transferrin, while SAT, iMg/tMg and iMg are negatively correlated. The second factor (F2) is positively correlated with serum Fe, tHgb, SAT, serum Mg; hematocrit, and negatively with iMg/tMg, and MetHgb. After 11 days of the study, F1 was determined with UIBC, TIBC, Transferrin; SAT; serum Fe; tHgb and hematocrit (-0.982, -0.890, -0.890, 0.890, 0.822, 0.777, and 0.698, respectively), whereas F2 was determined with iMg/tMg; iMg and serum Mg (0.981; 0.720 and -0.616; respectively), Figure 3B. The first factor (F1) is positively correlated with SAT; serum Fe; tHgb and hematocrit, and negatively with UIBC, TIBC and Transferrin, while second factor (F2) is positively correlated with iMg/tMg and iMg, and negatively with serum tMg. Table 4

**Table 4:** Associations between estimated O_2_Hgb and tertiles of whole blood ionized Mg at the eleventh day. p < 0.05 – a statistically significant difference for trend<br>T, tertil; O_2_Hgb, oxy-haemoglobin; N, number of participants

	Whole blood ionized Mg on the eleventh day			
Biochemical<br>parameter	T1 (N=17)<br><0.59	T2 (N=16)<br>0.60–0.63	T3 (N=13)<br>0.64–0.68	P for trend
O_2_Hgb (%)	49.9<br>(38.7–61.1)	41.3<br>(29.4–53.2)	27.3<br>(20.9–33.7)	0.029
				

## Discussion

To the best of our knowledge this is the first study examining the short-term effects of Mg supplementation in daily doses corresponding to 100% NRV on Fe status in young healthy women. Intervention study was conducted among young female subjects with an aim to explore the effects of short-term Mg supplementation on indices of iron status. Namely, the literature data indicate that the human body adapts to the additional intake of magnesium, calcium and phosphorus in the period from 7 to 10 days and that the mineral balance is achieved in the period of a few days after supplementation intake [29]. After 11 days of supplementation in a dose corresponding to Mg DRV (i.e. 375 mg), direct association was found between the serum tMg concentration and SAT. Furthermore, whole blood iMg correlated positively with SAT and serum Fe. These observations were also confirmed by polynomial regression. These finding suggest that Mg supplementation and increased Mg levels, both serum tMg and blood iMg, might exert beneficial impact on obtaining favorable Fe status among young female population.

In this sample, the average iron intake was significantelly bellow the DRI and DRV for women, i.e., 18 mg/day and 16 mg/day, respectively [4] [5]. These findings are consistent with the results of other studies which reported dietary iron intake in women of reproductive age in Europe [30] [31]. Regardless the suboptimal dietary intake of Fe, biomarkers of Fe status were within the normal range. This can be explained by the following facts: iron metabolism was primarily regulated at the level of absorption, and the examined population did not have gastrointestinal disorders. Also, the amount of iron that enters the body from food is regulated by the body's need for iron. Other studies also indicate that a significant proportion of the UK population has Fe intake below recommendations and a low prevalence of poor Fe status [32] [33] [34]. This might be because there are important uncertainties in the DRVs for Fe intake which may be too high, particularly for girls and women of reproductive age. It is recommended that the DRVs for iron should be reviewed when more data becomes available. Good quality dose-response data are required to enable a reassessment of the DRVs for iron. Knowledge of the systemic regulation and mediation of iron homeostasis should be applied to characterize better the responses to increased and reduced systemic needs for Fe and development, or better validation, of existing markers used to assess the adequacy of Fe status in populations and individuals. The main food sources of Fe among participants in our study were grains and cereal products with contribution of more than a quarter of total dietary intake of this nutrient followed by meat and meat products and vegetables. These three food groups together contributed to 65% of estimated iron intake in the participants. These observations are in accordance with previously published data for European population [35] [36]. Furthermore, dietary intake assessment revealed that Mg intake among female student population were also below the recommended level although mean baseline tMg concentration was adequate.

There is a lack of literature data regarding the effect of increased Mg intake on Fe status in young women in the reproductive period. This issue needs to be addressed taking into account that Mg supplements are one of the most popular dietary supplements used in adult population [17] and the fact that anemia is most prevalent among females of childbearing age [36].

In this study, we tried to explore the connection between serum Fe and serum Mg (as tMg, iMg and iMg/Mg) and other biochemical indices of Fe status using the PCA approach, before and after Mg supplementation. Given that the diet did not change over intervention period intake of Mg and Fe were not considered in PCA. At the beginning of the study, before the initiation of the supplementation, there was a strong positive correlation between UIBC, TIBC and transferrin, but after 11 days of supplementation we found a strong negative correlation among the same analyzed parameters. Analyses revealed that even in a short period of intervention there is a noticeable effect of Mg supplementation on Fe status parameters (serum Fe, tHgb, hematocrit). PCA analysis revealed a positive correlation between serum Fe and SAT after 11 days of supplementation. Reddy et al. have explained similiar associations in Fe status parameters but in patients with functional anemia in chronic kidney disease [37]. Anemia could be present as a latent condition, mostly in young women who are in the reproductive period. In order to optimize Fe status it is important to monitor biochemical parameters and routinely examine relevant markers [38].

Previously published data suggest an interaction between the resorption of divalent cations such as Mg and Fe. Namely, the deficiency of one divalent cation in the intestine can lead to increased resorption of other divalent cations [39]. In an animal model, it has been shown that Fe deficiency can lead to increase in intestinal absorption of Mg, calcium and phosphorus since the same receptor may be involved in the resorption of these chemically similar cations [40]. Low dietary intake of Mg in rats has been shown to increase Fe resorption [41]. Furthermore, *in vitro* studies have demonstrated that Mg salts can negatively affect absorption of Fe by raising the pH value as the availability of Fe salts in the intestinal tract is pH dependent [42]. Moreover, certain Mg salts can absorb Fe and thus interact with its absorption [42].

On the contrary, there are studies linking Mg intake and the risk of developing anemia [15] [16] [42] [43] [44]. In our study, we have demonstrated that short term supplementation with Mg and increased level of serum tMg and iMg could have benefical effects on% SAT and serum Fe. Magnesium is a cofactor of a large number of enzymes, with an important role in the synthesis of hemoglobin. Accordingly, Mg deficiency can interrupt hemoglobin synthesis and erythrocyte energy metabolism and result in anemia. In addition, Mg deficiency has been reported to be associated with an inflammatory process, which could lead to anemia [45]. Therefore, the question arises as to whether Mg supplementation is required in persons who are not deficient in this trace element, and leaves space to the future studies to examine longitudinal effects of Mg supplementation on Fe status indices.

The key limitation of the present study is missing data regarding the leukocyte, thrombocyte, and erythrocyte counts as well as ferritin level, after the intervention period although changes in these parametres couldn't be expected during short intervention period. Additionally, acknowledge that calculated transferrin concentrations provide limited information. Furthermore, the duration of the Mg supplementation may have been short to explore the dynamical changes of the supplements' effects on biomarkers of Fe status. Similar studies are of interest in the male population, as well.

## Conclusion

This study results indicate that Mg supplementation leads to an improvement in the certain iron status parameters even in individuals with optimal levels of these indices. Additionally, the analyzed parameters were significantly correlated, and after the intervention period, a significant positive association among the analyzed parameters was achieved. However, caution should be exercised when supplementing Mg, and laboratory monitoring of the interaction is required. Further research is warranted regarding the possible impact of the forms of Mg preparations that exist on the market, and whether they equally affect biochemical changes of iron status in healthy young people as well as in specific target groups of patients.

## Dodatak

### Acknowledgements

This work was supported by the Ministry of Education, Science and Technological Development of Serbia on the basis of contract No.175036 and No.451-03-68/2020-14/200161.

### Conflict of interest statement

All the authors declare that they have no conflict of interest in this work.
